# Complete genome sequence of fish-pathogenic *Aeromonas hydrophila* HX-3 and a comparative analysis: insights into virulence factors and quorum sensing

**DOI:** 10.1038/s41598-020-72484-8

**Published:** 2020-09-23

**Authors:** Lei Jin, Yu Chen, Wenge Yang, Zhaohui Qiao, Xiaojun Zhang

**Affiliations:** 1 Marine Fishery Research Institute of Zhejiang Province, Zhoushan, 316021 China; 2 Zhoushan Fishery Environments and Aquatic Products Quality Monitoring Center of Ministry of Agriculture China, Zhoushan, 316021 China; 3 College of Food and Pharmaceutical Sciences, Ningbo University, Ningbo, 315211 China; 4 Key Laboratory of Animal Protein Food Deep Processing Technology of Zhejiang Province, Ningbo University, Ningbo, 315211 China

**Keywords:** Gene expression, Genome, Genomics, Microbial genetics

## Abstract

The gram-negative, aerobic, rod-shaped bacterium *Aeromonas hydrophila*, the causative agent of motile aeromonad septicaemia, has attracted increasing attention due to its high pathogenicity. Here, we constructed the complete genome sequence of a virulent strain, *A. hydrophila* HX-3 isolated from *Pseudosciaena crocea* and performed comparative genomics to investigate its virulence factors and quorum sensing features in comparison with those of other *Aeromonas* isolates. HX-3 has a circular chromosome of 4,941,513 bp with a 61.0% G + C content encoding 4483 genes, including 4318 protein-coding genes, and 31 rRNA, 127 tRNA and 7 ncRNA operons. Seventy interspersed repeat and 153 tandem repeat sequences, 7 transposons, 8 clustered regularly interspaced short palindromic repeats, and 39 genomic islands were predicted in the *A. hydrophila* HX-3 genome. Phylogeny and pan-genome were also analyzed herein to confirm the evolutionary relationships on the basis of comparisons with other fully sequenced *Aeromonas* genomes. In addition, the assembled HX-3 genome was successfully annotated against the Cluster of Orthologous Groups of proteins database (76.03%), Gene Ontology database (18.13%), and Kyoto Encyclopedia of Genes and Genome pathway database (59.68%). Two-component regulatory systems in the HX-3 genome and virulence factors profiles through comparative analysis were predicted, providing insights into pathogenicity. A large number of genes related to the AHL-type 1 (*ahyI*, *ahyR*), LuxS-type 2 (*luxS*, *pfs*, *metEHK*, *litR*, *luxOQU*) and QseBC-type 3 (*qseB*, *qseC*) autoinducer systems were also identified. As a result of the expression of the *ahyI* gene in *Escherichia coli* BL21 (DE3), combined UPLC-MS/MS profiling led to the identification of several new N-acyl-homoserine lactone compounds synthesized by AhyI. This genomic analysis determined the comprehensive QS systems of *A. hydrophila*, which might provide novel information regarding the mechanisms of virulence signatures correlated with QS.

## Introduction

The motile gram-negative bacterium *Aeromonas hydrophila*, recognized as an opportunistic pathogen, has been widely found in a variety of aquatic environments and can infect fish, shrimps, crabs, and other aquatic animals, even possibly causing human diseases resulting from infections by consuming *A. hydrophila*-contaminated animals^1–5^. Motile aeromonad septicaemia (MAS) in different fish species is often involved in cases for which *A. hydrophila* is believed to be the causative agent^6^, and appears to be distributed globally. MAS disease outbreaks in the fish industry caused a yield reduction of approximately 30–40 thousand tons of fish per year between 1989 and 1993 in southeast China^7,8^, and led to an estimated economic loss of more than $3 million pounds in 2009 in the southeastern United States^9–11^. Hence, *A. hydrophila* linked to a variety of fish diseases has generated wide concern in the aquaculture industry.

The pathogenicity of *A. hydrophila* is mediated by multiple virulence factors, including haemolysin, cytotoxic enterotoxin, aerolysin, adhesins, the S-layer, exoprotease, lipase, elastase, the flagellum and lipopolysaccharides^12–15^. In fact, quorum sensing (QS), which regulates gene expression and diverse physiological changes, is usually considered to be the other player in the bacterial infection process^16,17^. Three typic autoinducer systems, the N-acyl-homoserine lactone (AHL)-based AI-1 system^18^, the S-ribosylhomocysteinase (LuxS)-based AI-2 system^19^, and the QseB/QseC-dependent autoinducer AI-3 system^20^ have been demonstrated to exist in *A. hydrophila* and to have positive or negative effects on biofilm formation, motility and virulence.

**Figure 1:**
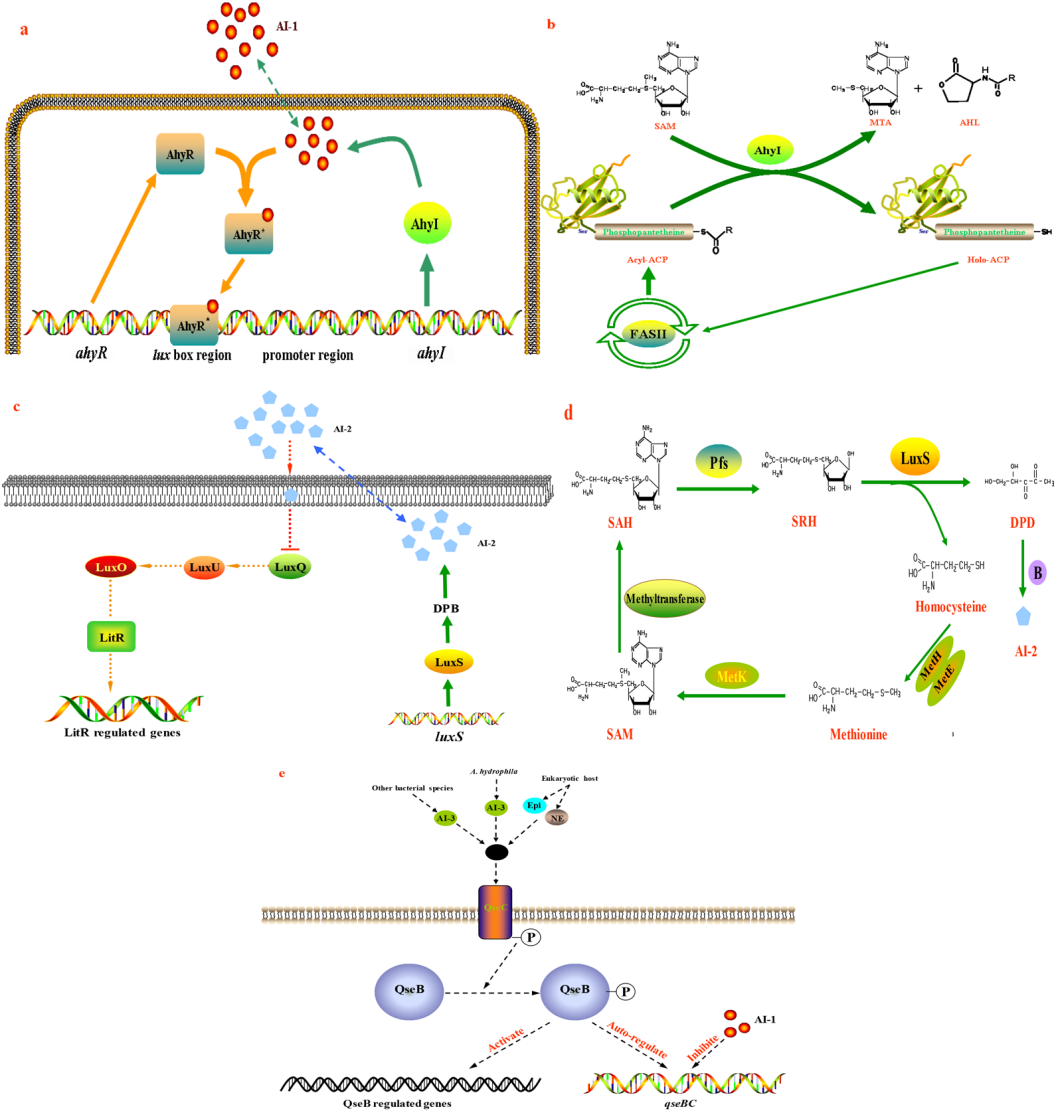
Models of the autoinducer AI-1, AI-2 and AI-3 quorum sensing system of *A. hydrophila*. (**a**) Schematic representation of QS regulation by AhyI/AhyR in *A. hydrophila*. AhyR^*^ represents the active AhyR. (**b**) Synthesis of AHL catalysed by AhyI protein using acyl-ACP and SAM. Fas II represents type II fatty acid synthesis reactions. (**c**) A putative regulatory mechanism of the LuxS-type 2 QS system in *A. hydrophila*. (**d**) AI-2 synthetic routes based on LuxS protein. DPD undergoes further reactions to form distinct biologically active signal molecules (AI-2), including (2*S*,4*S*)-2-methyl-2,3,3,4-tetrahydroxytetrahydrofuryl borate (*S*-THMF-borate) and (2*R*,4*S*)-2-methyl-2,3,3,4-tetrahydroxytetrahydrofuran (*R*-THMF). (**e**) A putative regulatory mechanism of the QseBC-based AI-3 system in *A. hydrophila*. The “P” surrounded by a circle represents phosphorylation.

The AI-1 QS system in *A. hydrophila* based on the AHL is composed of two core proteins, an AHL synthase (AhyI) and a transcriptional regulator (AhyR) encoded by the genes *ahyI* and *ahyR*, respectively^18,21^. A schematic diagram of the AI-1 quorum sensing system in *A. hydrophila* is presented in Fig. 1a. First, AhyI protein synthetizes AHL molecules, which freely diffuse across cellular membranes and accumulate in both the cell and surrounding environment^22^. When AHL concentrations exceed a threshold, they are recognized by the AhyR protein to form an AHL-AhyR complex that binds to the *lux* box region in the intergenic region 10 bp upstream of the *ahyI* promoter and induces the transcription of the downstream operon^23^. In a previous study, AhyI was recognized as an acyl-ACP-dependent AHL synthase that utilizes S-adenosyl-L-methionine (SAM) as an amino donor and acyl-acyl carrier protein (acyl-ACP) as an acyl donor to synthetize AHL and release methylthioadenosine (MTA) via acylation and lactonization reactions (Fig. 1b), while acyl-ACP is derived from fatty acid biosynthesis^24^.

To date, AI-2 synthetic pathways in *A. hydrophila* have been proposed (Fig. 1d)^25^. First, SAM is catalysed by methyltransferase to produce S-adenosylhomocysteine (SAH), and the degradation occurs by S-adenosylhomocysteine nucleosidase (Pfs) to form S-ribosylhomocysteine (SRH). Then, LuxS catalyses the cleavage of SRH into 4,5-dihydroxy-2,3-pentanedione (DPD), which spontaneously cyclizes to form a furanone and finally forms AI-2 molecules by possibly reacting with borate (-B). AI-2 molecules can freely diffuse across cell membranes and bind to a periplasmic receptor for indirect transcriptional regulation when the concentration reaches a threshold^26,27^. However, the AI-2 internalization step of *A. hydrophila* is not yet known (discontinuous traits). In the current study, a putative AI-2 quorum sensing system in *A. hydrophila* is presented in Fig. 1c. Transduction of the AI-2 signal and gene regulation in *A. hydrophila* may be involved in the phosphorylation cascade (-P) via LuxQ, LuxU, and ultimately LuxO, which subsequently activates the transcriptional regulator LitR, thereby repressing *lux* operon-related gene transcription^16^.

The AI-3 QS system involved in the regulation of flagella and motility was initially described in enterohaemorrhagic *E. coli*^28^, and contains two core components, the response regulator QseB and the sensor kinase QseC. AI-3 molecules are presumed to be one group of eukaryotic hormone-like signals (e.g. epinephrine, Epi, or norepinephrine, NE) associated with interkingdom cross-signalling, and are usually produced by other bacterial cells or other bacterial species^28,29^. Recently, this system was also found in *A. hydrophila*^20,30^, but the synthesis of AI-3 molecules is not yet known. A putative regulatory mechanism of the AI-3 QS system based on the *E. coli* autoinducer-3 model is presented in Fig. 1e^25^. First, QseC is activated by AI-3 signals and undergoes autophosphorylation (-P) and then transmits the signal by phosphorylation (-P) to the QseB. Finally, phosphorylated QseB activates the transcription of virulence-related genes and autoregulates the operon of *qseBC* genes.

Herein, a new member of *A. hydrophila* HX-3 isolated from *Pseudosciaena crocea* was identified with QS systems. Its complete genome was compared to those of seven closely related fish-epidemic *A. hydrophila* strains^31–36^, or other fully sequenced *Aeromonas* spp. More importantly, this study revealed a complete list of potential virulence-related genes and QS-related genes. Novel insights into the virulence factors/mechanisms of QS regulation contributing to the pathogenicity of *A. hydrophila* were investigated by functional genomic analysis.

## Materials and methods

### Bacterial strains and DNA extraction

*A. hydrophila* HX-3 was previously isolated from spoiled *Pseudosciaena crocea*^24^. Bacterial cultures of *A. hydrophila* HX-3 were grown in Luria–Bertani (LB) broth at 28 °C overnight, and then the genomic DNA of bacteria in the exponential growth phase was extracted using HiPure Bacterial DNA Kits (Magen, Guangzhou, China). The DNA concentration was detected using Qubit (Thermo Fisher Scientific, Waltham, MA, USA) and NanoDrop (Thermo Fisher Scientific, Waltham, MA, USA) instruments, and the integrity of the genomic DNA was analysed by 1% agarose gel electrophoresis.

### Whole-genome sequencing

The whole genome was sequenced by Illumina Hiseq combined with third-generation sequencing technology (Pacific Biosciences, Menlo Park, CA, USA). The high-quality genomic DNA of *A. hydrophila* HX-3 was fragmented with G-tubes and end-repaired to prepare SMRTbell DNA template libraries. Continuous long reads attained from single-molecule real-time sequencing runs (PacBio RS II) were used for de novo genome assembly in Falcon version 0.3.0^37^. A total of 133,453 raw reads with an average length of 8592.1 bp and total size of 1146.64 Mbp were obtained. Furthermore, qualified genomic DNA was converted to 300–400 bp insert-size Illumina libraries using the NEBNext Ultra DNA Library Prep Kit (NEB, USA). The libraries were sequenced on an Illumina HiSeq 4000 sequencer using paired-end technology (PE 150). Raw data generated from the HiSeq Illumina platform were filtered using a quality control analysis by FASTP version 0.20.0^38^. After filtering, clean reads were obtained by (1) removing reads with ≥ 10% unidentified nucleotides; (2) removing reads with ≥ 50% bases having phred quality scores ≤ 20; and (3) removing reads aligned to the barcode adapter. Then, the resulting clean reads were mapped to the *A. hydrophila* HX-3 genome sequences from the PacBio platform, and the genome assembly above was corrected using Pilon version 1.23^39^.

### Phylogenetic and comparative genomic analysis

Genome-wide comparisons of all 26 *Aeromonas* organisms were performed by OrthoMCL tools^40^ to identify orthologous genes, with a similarity cutoff of 30% and an E-value of 1e−5 using DIAMOND BLASTP alignments. The phylogenetic tree based on conserved core single-copy orthologous genes was constructed by Neighbor-joining method with 500 bootstrap replicates statistical support, using MEGA version X. The ANI values between two genome sequences were calculated by the Python module pyani (https://widdowquinn.github.io/pyani/)^41^. A heatmap based on ANI values was generated by using the package heatmap in statistical software R (version 3.6.3$2020). Pan-genome analysis was carried out using Panseq (https://github.com/chadlaing/Panseq)^42^. A Venn diagram of the unique/core gene content was generated with a custom pl script using the package VennDiagram.

### Functional annotations

Open reading frames (ORFs) were predicted using NCBI prokaryotic genome annotation pipeline^43^. The rRNAs, tRNAs and ncRNAs were predicted by using rRNAmmer (version 1.2)^44^, tRNAscan (version 1.3.1)^45^ and cmscan (version 1.1.2)^46^, respectively. Repeat elements such as interspersed repeat elements were identified by RepeatMasker (version 4.0.5)^47^, and tandem repeat elements were identified by TRF (version 4.09)^48^. Transposon prediction was carried out using TransposonPSI (version 20,100,822)^49^. Clustered regularly interspaced short palindromic repeats (CRISPR) elements were identified by CRISPRCasFinder (https://crisprcas.i2bc.paris-saclay.fr)^50^. Genomic islands (Gls) were determined with the web tool IslandViewer (version 4) using four independent methods IslandPick, SIGI-HMM, IslandPath-DIMOB and Islander^51^. Prophages were identified using the PHASTER web server (https://phaster.ca/)^52^. The genome sequence was annotated by BLAST against the NCBI non-redundant protein (Nr), Cluster of Orthologous Groups of proteins (COG), Gene Ontology (GO), Kyoto Encyclopedia of Genes and Genomes (KEGG), UniProt/Swiss-Prot, protein families (Pfam), Carbohydrate-Active enZymes (CAZy), Pathogen-Host Interactions (PHI), Virulence Factors of Pathogenic Bacteria (VFDB) and Comprehensive Antibiotic Resistance Database (CARD) databases. Two-component systems (TCSs) were predicted based on their structural characteristics^53^. Details of the Nr, COG, GO, KEGG and Swiss-Prot annotations can be found in Supplementary Information C. The web bTSSfinder server (https://www.cbrc.kaust.edu.sa/btssfinder) was used to predict promoter regions and Shine-Dalgarno (SD) sequences^54^. The complete genome sequence has been deposited in GenBank under accession number CP046954.

### Cloning and expression of the *ahyI* gene

The *ahyI* gene was amplified from genomic DNA of strain HX-3 using the following primer set: 5ʹ-CCGAATTCATGTTGGTTTTCAAAGGAAAATTAAAAGAAC-3ʹ (forward) and 5ʹ-TGCTCGAGTTATTCTGTGACCAGTTCGC-3ʹ (reverse). The PCR products with *EcoR*I and *Xho*I restriction sites (underlined) were cloned into pET-30a (+) to generate the recombinant plasmid pET30a-*ahyI*, and then transformed into *E. coli* BL21 (DE3). Kanamycin (20 μg/mL) was added to select the transformants. The AHL products of the screened transformants were detected using the *Chromobacterium violaceum* CV026 biosensor in response to short-chain AHLs with four to eight carbons and further analysed by UPLC-MS/MS.

### AHL extraction and detection

*E. coli* BL21 (DE3) with the recombinant plasmid pET30a-*ahyI* was cultured in 20 mL of LB broth supplemented with kanamycin (20 μg/mL) at 28 °C, induced with 0.1 mM isopropyl-β-D-thiogalactopyranoside (IPTG) at an OD600 of 0.6, and incubated for an additional 12 h at 28 °C. The AHL extraction method was performed as described previously^55^. UPLC-MS/MS analysis was carried out according to the methods described by Jin et al.^24^, with slight modification. A detailed description of the UPLC-MS/MS methods is presented in Supplementary Information A “Supplementary Methods”.

## Results and discussion

### Genomic features of *A. hydrophila* HX-3

**Figure 2:**
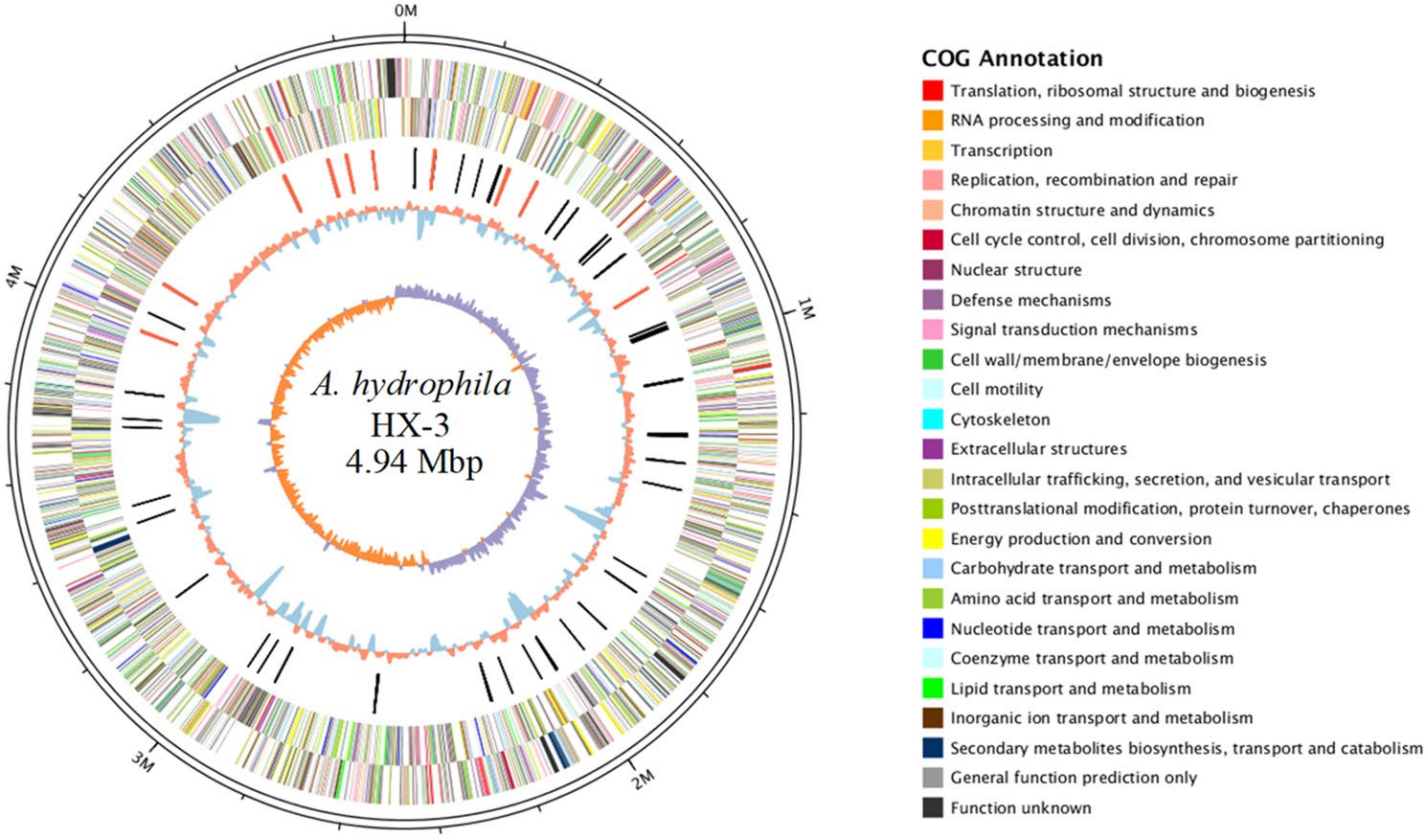
Circular map for the whole genome of *A. hydrophila* HX-3. From the outside to the centre: genome sequence coordinates, genes encoded on forward and reverse chains (different colours based on COG categories), ncRNA (red: rRNA, black: tRNA), GC content, and GC skew (G − C/G + C).

The complete genome of *A. hydrophila* HX-3 comprises one circular chromosome of 4,941,513 bp with a G + C content of 61.0% (Fig. 2). The genome contains 4483 predicted genes, of which 4318 are coding DNA sequences (CDSs). No plasmids were found during the HX-3 genome analysis. Previous studies have shown that the antimicrobial resistance and virulence in pathogenic *A. hydrophila* may be plasmid-mediated. For instance, a 21-kb plasmid plays a pivotal role in the specific virulence and pathogenicity of *A. hydrophila* VB21 when injected in *Clarias batrachus*^56^. A 165,906-bp circular plasmid, pR148, confers on *A. hydrophila* isolates the antimicrobial resistance against streptomycin, chloramphenicol, mpicillin, tetracycline, and sulfamonomethoxine^57^. However, plasmids as the mobile elements were absent in the HX-3 genome sequence, which indicated that they could be dispensable elements to impact the overall environmental fitness and virulence in a plasmid-negative *A. hydrophila* infection.

**Figure 3:**
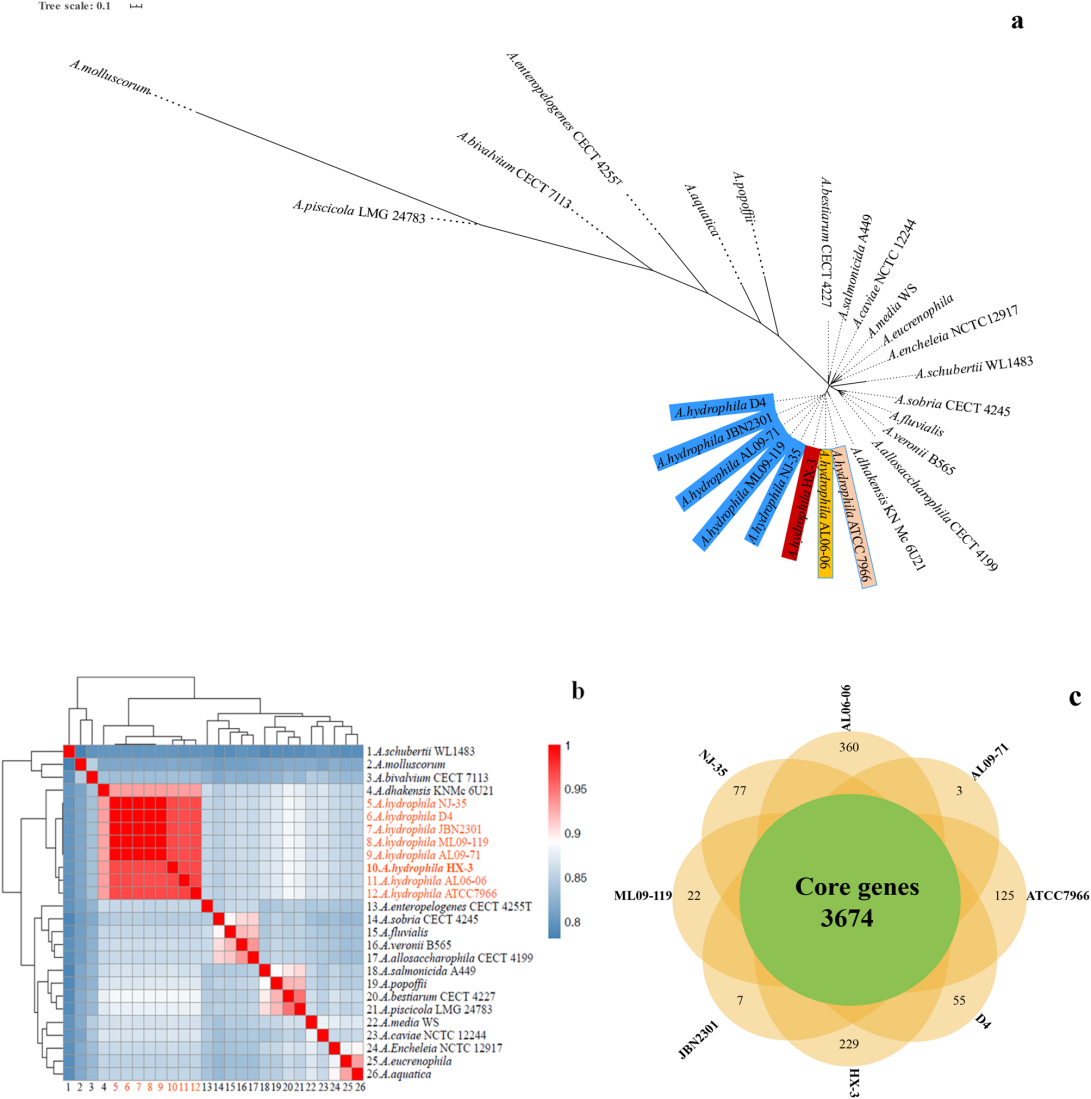
Genomic comparison of the *A. hydrophila* HX-3 with other *Aeromonas* species. GenBank assembly accession for 26 strains showed in the Supplementary Information B “ANI values”. (**a**) Phylogenetic tree constructed based on conserved core single-copy orthologous genes of 26 *Aeromonas* strains complete genomes. Eight *A. hydrophila* strains are colored in blue, red and yellow, respectively. The same color indicates *A. hyrophila* members clustered on a branch. (**b**) Heatmap of 26 strains based on the average nucleotide identity (ANI) values between two *Aeromonas* species. (**c**) Venn diagram of core and unique genes shared by comparison of eight closely related *A. hydrophila* strains.

Comparative analysis of HX-3 genome and 25 fully sequenced *Aeromonas* species genomes was performed to confirm the evolutionary relationships. A phylogenetic tree constructed based on conserved core single-copy orthologous gene alignment showed the clear lineage divergence, and *Aeromonas* species all formed monophyletic branches (Fig. 3a). Four terminal branches were clearly separated for eight virulent *A. hyrophila* strains. Further, five *A. hyrophila* members (NJ-35, ML09-119, AL09-71, JBN2301 and D4) clustered together with a high bootstrap value support (100%), which suggested that they had a close evolutionary relationship. *A. hyrophila* HX-3 formed an independent branch and was distinct from other seven *A. hyrophila* isolates tested. In order to further confirm evolutionary relationship, ANI phylogenetic analysis was performed to estimate genomic differences and relatedness between two genomes. As a result (Fig. 3b and Supplementary Information B “ANI values”), the genomes of eight *A. hydrophila* strains shared ANI values ranging from 96.64 to 99.99%, which are values above the threshold of 94–96% identity usually used to serve as a speciation boundary^58^. On the other hand, the *A. hydrophila* HX-3 genome was found to share identities with the other seven *A. hyrophila* strains of the ANI group of less than 97%, which indicated that HX-3 appeared to be a distinct strain from those currently classified within the *A. hydrophila* group.

As shown in the Venn diagram constructed for 8 closely related *A. hyrophila* genomes (Fig. 3c), the pan-genome consisted of 5778 genes, including 3674 core genes (63.59%), 1226 accessory genes (21.22%), and 878 unique genes (15.19%). Variability in the number of unique genes was observed from 3 to 360 genes, and the least and most number of unique genes were identified in *A. hyrophila* AL09-71 and AL06-06, respectively. Notably, the strain HX-3 contains 229 unique genes and shares higher number of core genes with strains D4 (3918), ML09-119 (3902) and JBN2301 (3889). These results confirmed the high conservation and diversity of genome structure of the eight virulent *A. hyrophila* strains. Additionally, RNAmmer predicted HX-3 contains ten rRNA operons (encoding 11, 10, and 10 copies of 5S rRNA, 16S rRNA, and 23S rRNA genes, respectively) consistent with those in the genomes of seven other *A. hydrophila* strains (Table 1). A total of 127 tRNA genes were predicted by tRNAscan-SE, equal to the numbe in the genome of *A. hydrophila* ATCC 7966^T^. Intriguingly, the numbers of tRNAs in strain HX-3, ATCC 7966^T^ and JBN2301 were clearly higher than those in the other five strains. There are at least two copies of tRNA genes for each specific amino acid, and the tRNA genes with the highest copy number of thirteen for methionine are encoded in the genome of *A. hydrophila* HX-3. Based on comparisons with the Rfam database, a total of 7 non-coding RNAs (ncRNAs), otherwise known as regulatory RNAs, were predicted by the software cmscan, consistent with the results for the genomes of *A. hydrophila* D4 and ML09-119, except that more were obtained than in the other four strains.

**Table 1:** General features of eight *A. hydrophila* genomes.

Strain	HX-3	D4	AL09-71	AL06-06	JBN2301	ML09-119	NJ-35	ATCC7966^T^
Accession number	CP046954	CP013965	CP007566	CP010947	CP013178	CP005966	CP006870	CP000462
Source	Pseudosciaena crocea	Blunt-snout bream	Channel catfish	Channel catfish	Crucian carp	Channel catfish	Carp	Milk
Length (bp)	4,941,513	5,100,520	5,023,861	4,384,823	5,127,362	5,024,500	5,279,644	4,744,448
G + C content (%)	61.00	60.80	60.80	61.30	60.77	60.80	60.51	61.50
No. of genes	4483	4774	4492	4472	4646	4698	4716	4284
No. of CDS	4318	4619	4297	4251	4438	4548	4526	4119
No. of 5S rRNAs	11	11	11	11	11	11	11	11
No. of l6S rRNAs	10	10	10	10	10	10	10	10
No. of 23S rRNAs	10	10	10	10	10	10	10	10
No. of tRNAs	127	117	111	112	129	112	102	127
No. of ncRNAs	7	7	2	4	1	7	2	Unclear

Genetic repeat elements (interspersed or tandem repeats) are components of gene regulatory networks and have diverse functional roles in evolution, heredity and variation, such as mismatch repair and damage repair of nucleic acid bases^59^. DNA replication origins for many bacteria and viruses often contain direct repeat, palindrome and simple repeat sequences^60^. For the repeat element analysis, the results (Fig S2) showed that the repeat elements identified in *A. hydrophila* HX-3 constituted 1.18% of the whole genome, including 0.20% as interspersed repeat sequences and 0.98% as tandem repeats. Among the interspersed repeats, 47 SINEs (short interspersed nuclear elements), 17 LINEs (long interspersed nuclear elements) and 6 DNA elements were identified, while no LTR (long terminal repeat) or unclassified elements were detected. Among the tandem repeats, a total of 153 repeats were identified, including 146 minisatellite DNA repeats and 7 microsatellite DNA repeats, but no satellite DNA was found.

A total of 7 types of transposons were identified in the *A. hydrophila* HX-3 genome, including 4 full-length Ty1/copia retrotransposons, 2 Ty3/gypsy LTR-retrotransposons and 1 LINE retrotransposon (see Supplementary Information B “Transposon”). Moreover, the strain HX-3 genome contains 8 CRISPR loci with a total of 15 spacers and only one CRISPR-associated (*cas*) gene (*cas5/casD*, GQR50_07335), which could confer resistance against the intrusion of mobile elements such as phages and plasmids^61^ (Supplementary Information B “CRISPR”). A total of 39 GIs with 385 genes were detected in the genome (Supplementary Information B “Gls”). In the GIs, 20 encoded transposases belonged to the IS5, IS630, IS66, IS3, IS5/IS1182, and IS1595 families, and four belonging to GL_017. A total of 116 and 18 genes were identified that encoded hypothetical proteins and tyrosine-type recombinase/integrase, respectively. Two genes in GL_024 and GL_032 were predicted to encode the T6SS proteins VgrG and one in GL_009 was predicted to encode another T6SS effector, Hcp1. Another two genes in GL_006 and GL_037 were predicted to encode T2SS-secreted toxins. Clearly, no prophage sequences were identified.

### Gene functional analysis

**Figure 4:**
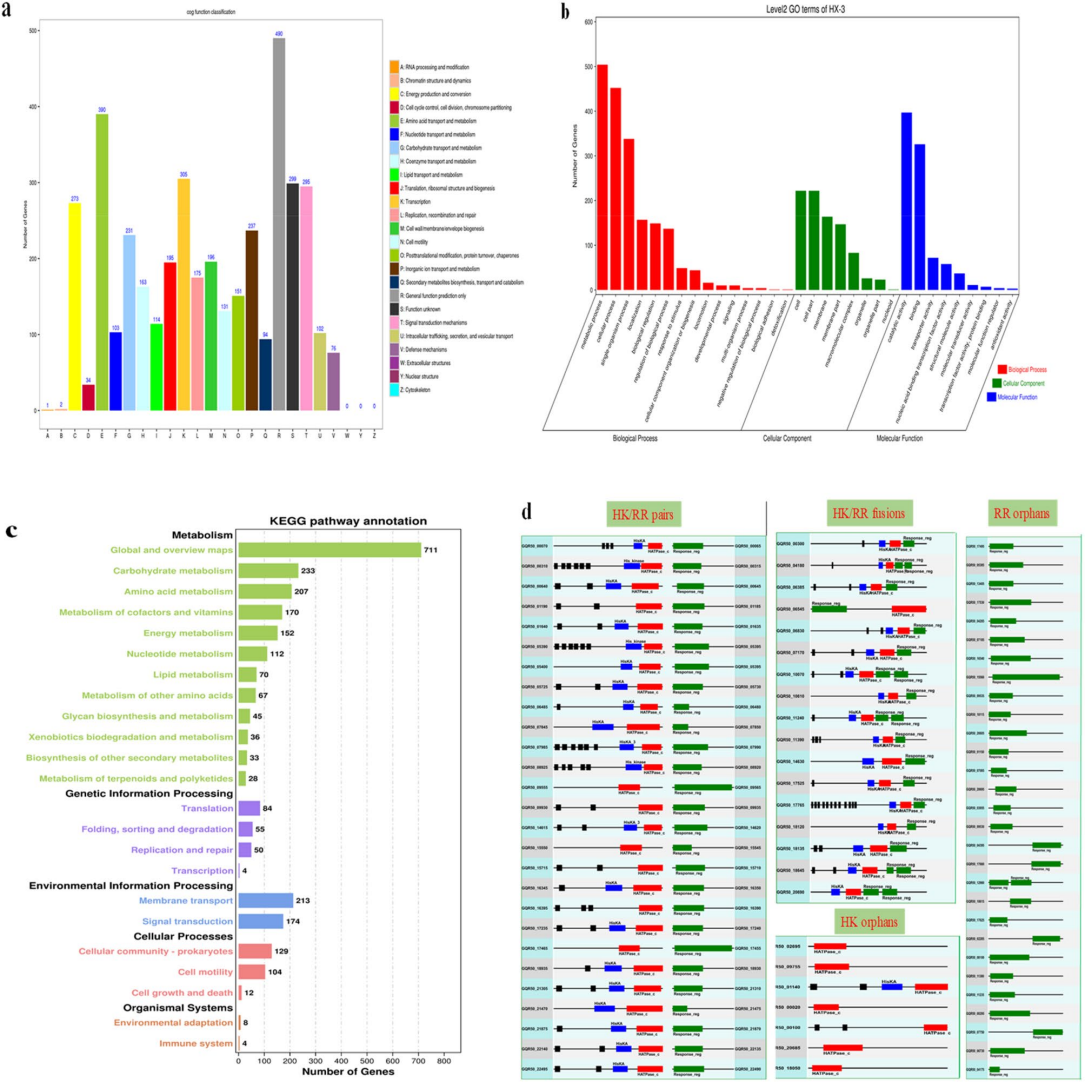
Gene functional annotation of the *A. hydrophila* HX-3 genome in the COG, GO and KEGG databases. (**a**) COG annotation classifications. The COG functional annotations were divided into 25 categories. The COG categories are shown on the X-axis as alphabets, with category names on the right. (**b**) GO annotation distribution (level 2). The GO assignments were divided into three categories (level 1) namely, biological process (red), cellular process (green), and molecular function (blue). (**c**) KEGG annotation distribution. The KEGG orthologies were categorized into five major categories: metabolism (green), genetic information processing (purple), environmental information processing (blue), cellular processes (pink), and organismal systems (orange). (**d**) Genes encoding two-component systems in *A. hydrophila* HX-3. TCSs could be categorized into four groups: HK/RR protein pairs (27 gene pairs), HK/RR fusion (17 genes), HK orphans (7 genes) and RR orphans (29 genes).

The COG categories were generated by comparing predicted and known proteins in all completely sequenced genomes of *Aeromonas* and other bacteria to infer sets of orthologues. The results revealed that a total of 3283 genes were annotated in the COG database and distributed in 22 different functional categories (from A to Z, Fig. 4a). In the COG classification, the five most abundant annotated functions were as follows: (1) general function, encoded by 490 genes, accounting for 12.8% of the total functional annotations; (2) amino acid transport and metabolism function, with 390 classified genes, accounting for 9.61%; (3) transcription function, with 305 classified genes, accounting for 7.52%; (4) signal transduction mechanism function, with 295 classified genes, accounting for 7.27%; (5) energy production and conversion function, with 273 classified genes, accounting for 6.73%. Furthermore, 299 unique genes accounting for 7.37% of the total annotations were classified as having unknown functions, which may be revealed by further functional studies. In addition, only one gene and two genes were involved in RNA processing and modification, and chromatin structure and dynamics, respectively. Genes related to extracellular structure, nuclear structure, and the cytoskeleton were not detected in the genome of HX-3.

Gene Ontology (GO) is a functional classification system regarding the functions of genes and gene products. In this study, 783 protein-coding genes were categorized by GO analysis (Fig. 4b) (for details, see Supplementary Information B “GO”). The differentially expressed genes were mainly found in 32 subfunctional items of three major categories: biological process (15 subfunctions), cellular composition (8 subfunctions) and molecular function (9 subfunctions). A total of 1876 differentially expressed genes were annotated as belonging to the biological process category, with most involved in metabolic process (GO:0008152), cellular process (GO:0009987), and single-organism process (GO:0044699). A total of 888 genes were annotated as belonging to the cell component category, with most involved in the subfunctions cell (GO:0005623) and cell part (GO:0044464). A total of 915 genes were annotated as belonging to the molecular function category, of which the differentially expressed genes with the subfunctions catalytic activity (GO:0003824) and binding (GO:0005488) were the most numerous. Altogether, genes annotated to the subfunctions metabolic process, cellular process and catalytic activity were the most numerous.

The KEGG database is an integrated database resource for the functions of gene products and cell metabolic pathways. A total of 2577 genes were annotated in the KEGG orthology (KO) database and divided into five categories: metabolism (1864 genes), genetic information processing (193 genes), environmental information processing (387 genes), cellular processes (245 genes), and organismal systems (12 genes) (Fig. 4c). The KO distribution results showed that the most abundant orthology was global and overview maps from the “Metabolism” category, with 711 genes. The second most abundant was carbohydrate metabolism from the “Metabolism” category (233 genes), followed by membrane transport (213 genes) and amino acid metabolism (207 genes) from the “Environmental information processing” and “Metabolism” categories, respectively. In epidemic *A. hydrophila* ST251^62^, three carbohydrate metabolic pathways utilizing *myo*-inositol, sialic acid and L-fucose are predicted to be the key factors causing the strain to be epidemic by helping it overcome nutritional limitations in vivo and thereby increasing its fitness during infection. However, only two specific metabolic pathways utilizing *myo*-inositol (GQR50_13900) and sialic acid (GQR50_20745) were identified in our study, which indicated that *A. hydrophila* HX-3 exhibits less virulence than the clinical isolates.

The analysis of the genome suggests that *A. hydrophila* HX-3 responds efficiently to environmental changes due to the encoded systems for gene expression regulation, such as bacterial two-component systems (TCSs), composed of a sensor histidine kinase (HK) and a response regulator (RR)^63–66^. Upon signal perception, the HK first autophosphorylates on a histidine residue and then transfers the phosphoryl group to the cognate RR, which binds target promoters and thereby regulates gene expression^67,68^. In the genome of *A. hydrophila* HX-3, a total of 54 genes encode twenty-seven HK/RR protein pairs and 17 genes encode the independent HK/RR fusion proteins (Fig. 4d). In addition, single histidine kinase and response regulator proteins are encoded by 7 and 29 genes, respectively. TCSs are often the critical regulators of pathogenicity and virulence in *A. hydrophil*a. For instance, one TCS (QseB/QseC) can modulate the in vitro and in vivo virulence of *A. hydrophil*a by affecting motility, protease production, exotoxin secretion and biofilm formation^20^. The PhoP/PhoQ two-component system that mediates adaptation to Mg^2+^-limiting environments has a negative regulatory effect on the expression of T3SS and virulence factors^69^.

### Virulence factor analysis

The type II secretion system (T2SS) is a major virulence factor, responsible for the extracellular secretion of protein toxins and degradative enzymes that mediate pathogenic effects^70,71^. Several T2SS component genes encoding ExeAB (GQR50_02545-02550), ExeN-C (GQR50_19875-19935) and TapD (GQR50_02110) were identified in the genome of *A*. *hydrophila*, and these genes were also present in seven other *A. hydrophila* strains (Table 2). The type III secretion system (T3SS) utilized by some gram-negative bacteria to inject effector proteins into the cytosol of host cells^72^ was present only in strain AL06-06, which indicates that the T3SS makes a small contribution to the virulence of *A*. *hydrophila* HX-3 and other strains. Conversely, a functional type VI secretion system (T6SS) with several T6SS effectors^73^, including three haemolysin-coregulated proteins (Hcp, ID: GQR50_04035, GQR50_13180, GQR50_17010), three valine-glycine repeats G (VgrG, ID: GQR50_13070, GQR50_13175, GQR50_17005) and one proline-alanine-alanine-arginine repeat (PAAR, ID: GQR50_13075), was identified in the *A. hydrophila* HX-3 genome (Table 2). In contrast to the T3SS, a gene cluster encoding a T6SS that was present in 7 of the 8 *A. hydrophila* genomes (except for strain AL09-17) was more widely distributed, which suggested that T6SS can play a significant role in the pathogenicity of these strains.

**Table 2:**
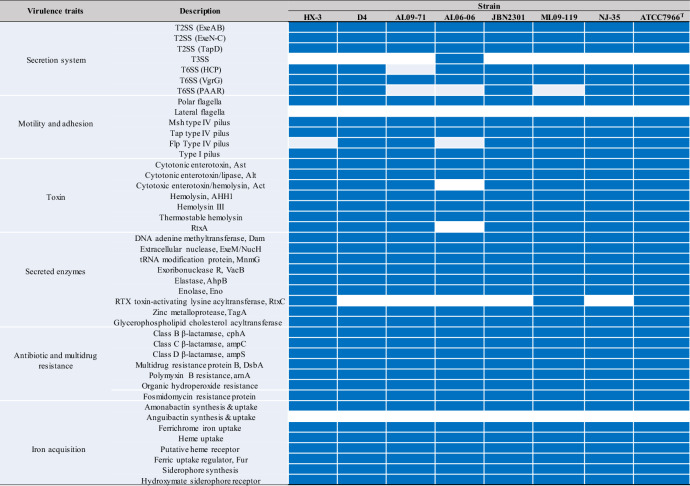
Distribution of potential virulence genes in eight *A. hydrophila* genomes.

The cells coloured blue and white indicate gene presence and absence, respectively.

**Figure 5:**
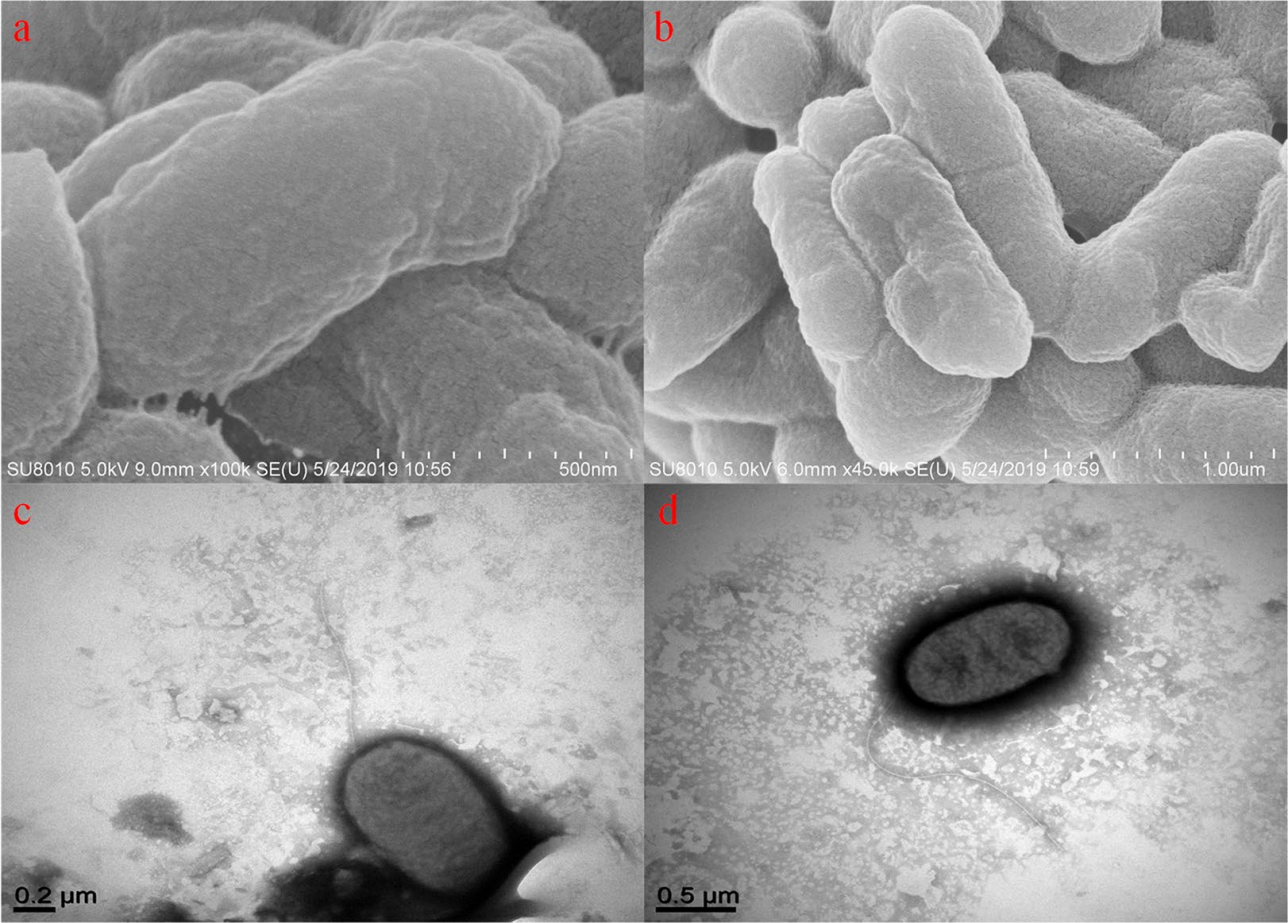
Electron micrograph of *A. hydrophila* HX-3: SEM (**a**, **b**) and TEM (**c**, **d**).

The role of motility and adhesion in *A. hydrophila*, enabled by flagella or pili, may facilitate the invasion of fish cell lines^74,75^. Genes for the polar flagella were found in four gene clusters dispersed throughout the genome of HX-3 (GQR50_07735-07810, 07840–07850, 15535–15655, and 15575–15655), which had the same distribution pattern in the other seven strains, whereas lateral flagella were absent in all strains. Both TEM and SEM analysis (Fig. 5) confirmed that *A. hydrophila* HX-3 possessed a single polar flagellum and lacked lateral flagella. The genes for three different pili, including the Msh type IV pilus (GQR50_20800-20880), Tap type IV pilus (GQR50_02125-02110, 03235-03240, 05870-05890, 08610, 13680, and 03160) and Type I pilus (GQR50_20150-20175), were found in the *A. hydrophila* HX-3 genome, and was present in all eight genomes. Interestingly, the Flp type IV pilus was absent in *A. hydrophila* HX-3 and AL06-06. In contrast to the Tap pilus, the Flp type IV pilus was confirmed to make little or no contribution to the virulence of *Aeromonas* species against Atlantic salmon, while the Flp pilus is an important factor for adherence to host surfaces^76^.

Other classes of virulence factors are toxins and secreted enzymes, which are also associated with the pathogenic potential of *A. hydrophila*^62,75,77,78^. Several typical toxin-encoding genes were identified in the *A. hydrophila* HX-3 genome, including the cytotoxic enterotoxin Act (GQR50_20590), the two cytotonic enterotoxins Ast (GQR50_18705) and Alt (GQR50_22295), haemolysin AHH1 (GQR50_14970), haemolysin III (GQR50_04325), thermostable haemolysin (GQR50_05755) and RtxA (GQR50_15680). The *act* and *rtxA* genes were absent in the AL06-06 genome. In addition, a number of genes encoding secreted enzymes were also detected in the *A. hydrophila* HX-3 genome and apparently conserved in all of the genomes of *A. hydrophila*; however, the RTX toxin activator gene *rtxC* found in the *A. hydrophila* HX-3 genome (GQR50_15685) was absent in the genomes of *A. hydrophila* D4, AL09-71, AL06-06, JBN2301 and NJ-35.

Antibiotic and multidrug resistance, two additional classes of virulence factors^75,77,79^, were well represented in the *A. hydrophila* HX-3 genome and distributed similarly among all of the *A. hydrophila* strains. Three β-lactamase genes, *ampC* (GQR50_06175), *ampS* (GQR50_22500) and *cphA* (GQR50_19040), are located on the HX-3 chromosome that also carries genes encoding DsbA (GQR50_21040), polymyxin B ArnA (GQR50_17695), organic hydroperoxide (GQR50_03510-03520) and fosmidomycin (GQR50_00245) resistance proteins. Genes for several multidrug efflux systems were also identified, including members of the RND (GQR50_00105, 07065), DMT (GQR50_10960), SMR (GQR50_03945), MFS (GQR50_09275, 11,130, 13,885, 18,395), and ABC (GQR50_22590) superfamilies.

Iron acquisition is typically recognized as an essential factor for bacterial pathogen survival in the host, and it significantly contributes to *A. hydrophila* virulence and is a key factor for infectious fish disease development^80^. In the genome of *A. hydrophila* HX-3, the genes involved in the synthesis and uptake of amonabactin (well known as a phenolate siderophore) were located within a cluster (GQR50_09810-09840), which confers to the HX-3 isolate the capacity to obtain iron for growth during a systemic *A. hydrophila* infection^81^. The amonabactin synthesis gene cluster is widespread in other *Aeromonas* species, such as the fish pathogen *A. salmonicida*^82^, and importantly, the latest study by Balado et al*.*^83^ revealed the biosynthetic pathway for amonabactin in *A. salmonicida* subsp *salmonicida*. However, genes encoding the synthesis and uptake of anguibactin were not found in any of the *A. hydrophila* strains. An additional gene cluster for siderophore synthesis (GQR50_05430-05435) was characterized in the HX-3 genome and a gene cluster (GQR50_22585-22605) encoding a hydroxamate siderophore receptor and an ABC transporter system, indicated that HX-3 may use a hydroxamate-type ferric siderophore for iron acquisition. Genes for ferrichrome iron uptake (GQR50_12415-12430), haeme uptake (GQR50_17805-17825), haeme receptor (GQR50_17785), and a ferric uptake regulator (GQR50_14875) were identified in the genomes of HX-3 and other strains.

### Quorum sensing system in *A. hydrophila*

QS is a bacterial communication system involving the secretion and detection of specific signal molecules to regulate gene expression and diverse physiological changes^84,85^. Recent studies have focused on the relationship between pathogenicity and QS in *A. hydrophila*. Three different QS systems have been described in *A. hydrophila*, including the AHL-type 1, LuxS-type 2 and QseBC-type 3 autoinducer systems^18–20^.

#### Type 1 autoinducer (AI-1) system

Based on the genome analysis, the core genes of the type I QS system encoding AhyR protein (GQR50_19990) and AhyI protein (GQR50_19995) were identified in the HX-3 genome, and the *ahyI* gene (red arrow) was located downstream of the *ahyR* gene (blue arrow) in reversed orientation, with an intergenic region of 62 bp (Fig S3b). The *ahyI/R* and adjacent genes were analysed in comparison with those of the other members of *A. hydrophila*. The analysis showed that these genes were distributed similarly among all of the *A. hydrophila* strains and highly homologous to each gene of strain HX-3 (Fig S3a). Based on nucleotide sequence alignment, *ahyI* and *ahy*R of strain HX-3 shared 99.04% and 99.36% identity with the homologous genes of *A. hydrophila* ATCC 7966^T^, respectively. In addition, a promoter region (highlighted in red) located at the − 29 bp central position upstream of *ahyI* was predicted, and a *lux* box sequence (highlighted in green) was identified at position − 52 to − 41 bp position based on the conserved sequences upstream of the promoter region (Fig S3b)^23^. An SD sequence (highlighted in the underlined bases) was also predicted to be responsible for transcription of *ahyI*.

**Figure 6:**
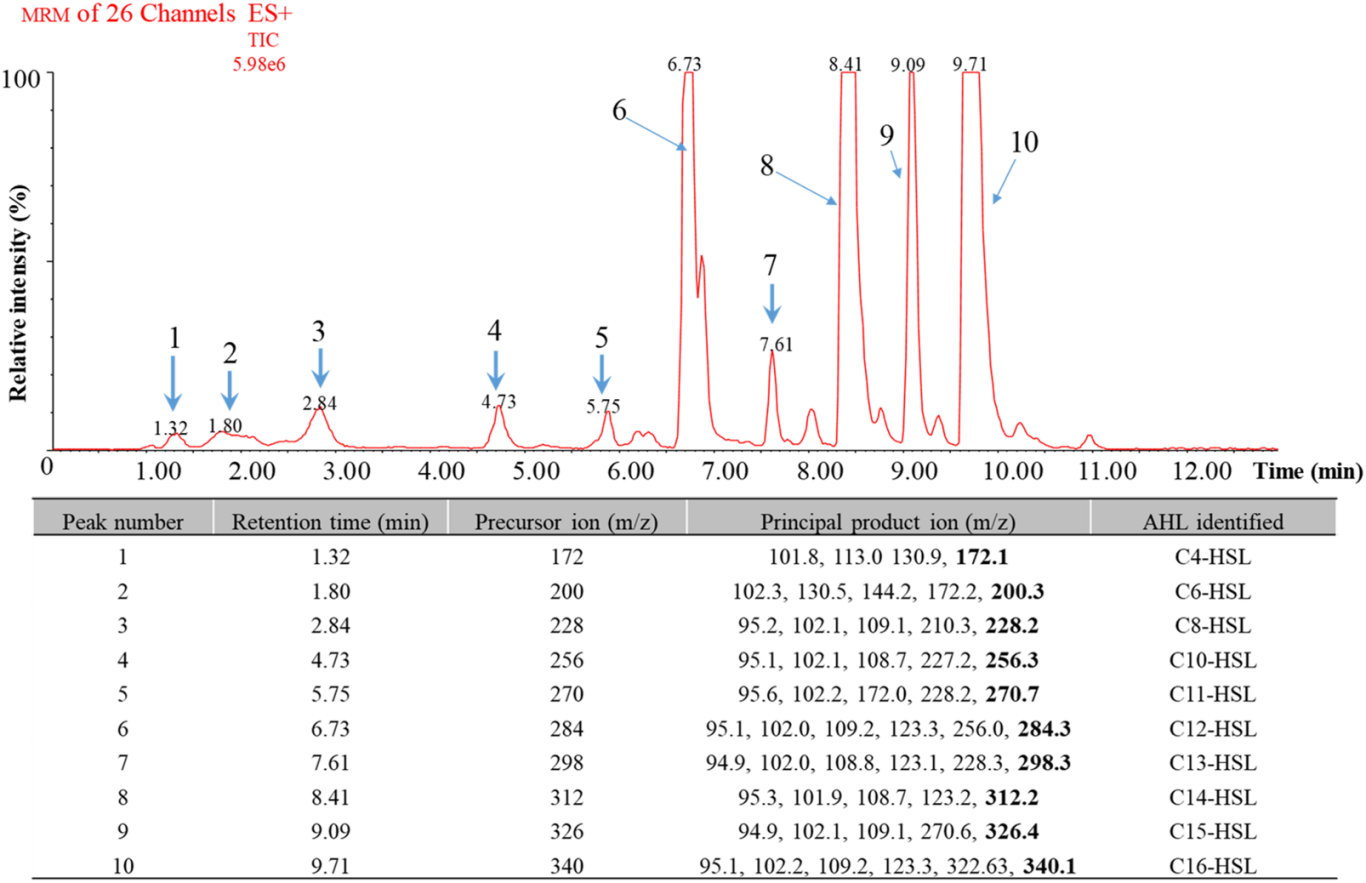
Total ion current (TIC) UPLC-MS/MS chromatograms of extracts from recombinant *E. coli* BL21 (DE3) harbouring pET30a-*ahyI*. The identified compounds with retention times and principal product ions are listed in the table.

To assess the dominant AHL products of the enzyme AhyI, *E. coli* BL21 (DE3) harbouring pET30a-*ahyI* was grown in LB medium (kanamycin, 20 μg/mL), and the supernatant extracts were analysed by UPLC-MS/MS*.* Previous studies confirmed that an increase in retention can be observed as the acyl-chain length increases^86,87^, which was used to predict the unknown compounds in combination with MS data. Thus, a total of 10 types of AHL compounds were identified based on the retention time and precursor ion *m/z* 102, and Fig. 6 shows predominantly the following ions: *m/z* 172 for C_4_-HSL, *m/z* 200 for C_6_-HSL, *m/z* 228 for C_8_-HSL, *m/z* 256 for C_10_-HSL, *m/z* 270 for C_11_-HSL, *m/z* 284 for C_12_-HSL, *m/z* 298 for C_13_-HSL, *m/z* 312 for C_14_-HSL, *m/z* 326 for C_15_-HSL and *m/z* 340 for C_16_-HSL (mass data shown in Supplementary information A “Fig. S1”). In terms of AHL profiles, diverse acyl-chain lengths of AHLs (C_4_ ~ C_14_-HSLs) and the level of saturation and side-chain modification by 3-oxo substituents have been observed in *Aeromonas* species in culture supernatants^24,25^. However, most *A. hydrophila* were observed to produce C_4_-HSL, C_6_-HSL or both^25^, and other members isolated from milk produce three types of HSL, C_8_-HSL, C_12_-HSL and C_14_-HSL^88^. Moreover, AhyI proteins from *A. hydrophila* have been previously verified to produce only two types of short-chain AHLs, i.e., C_4_-HSL and C_6_-HSL^21^. In this study, a transformant harbouring pET30a-*ahyI* was shown to produce 10 types of short- and long-chain AHLs, and the results may indicate the high flexibility of the choice of acyl-ACP utilized by AhyI, consistent with the other AHL synthases RhlI^89^, BmaI1^90^ and BjaI^91^ using *E. coli* ACP as acyl substrates.

For many years, the functions of AI-1 in *A. hydrophila* have been studied by constructing *ΔahyI* and *ΔahyR* mutants. As shown in Table 3, mutagenesis studies demonstrated that the AI-1 QS system influences the expression of numerous virulence factors in *A. hydrophila*, including the production of exoprotease, haemolysin, amylase, DNase, the S-layer, and T6SS-associated effectors (Hcp, VgrG) and biofilm formation. Interestingly, many studies reported that haemolysin is upregulated by the AI-1 QS system^93,96^, but other studies observed downregulation of the production of haemolysin^18,95^. Overall, AI-1 system-associated genes and AHL compounds produced by AhyI were identified in the present study, suggesting that the AhyRI solo system in *A. hydrophila* HX-3 plays an important role in pathogenic potential. However, the evidence for the underlying mechanism between the AI-1 QS system and virulence factors in *A. hydrophila* needs to be reinforced because of conflicting data, i.e., haemolysin production.

**Table 3:** Influence of three autoinducer (AI-1, AI-2 and AI-3) QS systems on the expression of virulence factors in *A. hydrophila*.

Autoinducer system	Virulence factors	Effect	Strains	Mutants	References
AI-1	Exoprotease	+	*A. hydrophila* AH-1N	Δ*ahyI* and Δ*ahyR*	^18^
	β-Haemolysin	−			
	Biofilm	+		*ΔahyI* and *ΔahyR*	^92^
	Proteases, amylase, Dnase, haemolysin and S-layer	+	*A. hydrophila* J-1	*ΔahyR*	^93^
	T6SS(Hcp, VgrG), metallo-protease, biofilm	+	*A. hydrophila*{*A. dhakensis*} SSU	*ΔahyRI* and *ΔahyR*+	^17,94^
	T3SS	+	*A. hydrophila*{*A. piscicola*} AH-3	*ΔahyI* and *ΔahyR*	^69^
	Haemolysin	−	*A. hydrophila* ATCC 7966	*ΔahyI*, *ahyI-K7Q* and *ahyI-K7R*	^95^
	Protease	+			
	Biofilm, proteases, haemolysin, amylase and Dnase	+	*A. hydrophila* YJ-1	*ΔahyI*	^96^
AI-2	Swimming motility	+	*A. hydrophila *{*A.dhakensis*} SSU	*ΔluxS*	^19^
	Biofilm	−			
	Biofilm, extracellular protease	+	*A. hydrophila* ATCC 7,966	*ΔluxS*	^97^
AI-3	Motility, cytotoxic enterotoxin, haemolysin, protease	+	*A. hydrophila* {*A. dhakensis*} SSU	*ΔqseB*	^20,94^
	Biofilm	−			
	Biofilm, haemolysin	+	*A. hydrophila* NJ-35	*ΔqseC*	^98^

“+” represents upregulation, and “−” represents downregulation.

#### Type 2 autoinducer (AI-2) system

The genomic analysis of *A. hydrophila* HX-3 revealed core genes predicted to be involved in the AI-2 synthesis of the strain. These genes encoded MtnN (homologue of Pfs, GQR50_13920), LuxS (GQR50_19260), MetH (GQR50_20625), MetE (GQR50_12405), MetK (GQR50_06220) and SAM methyltransferase (GQR50_12270), which suggested that *A. hydrophila* HX-3 could produce AI-2 through the LuxS solo system. In addition, genes for AI-2-related QS regulation were present in the genome of *A. hydrophila* HX-3. GQR50_03840 encodes a transcriptional regulator of the LitR family, probably controlling the expression of LitR-regulated genes, and GQR50_14630, GQR50_14645 and GQR50_17625 encode three putative signal transduction proteins (AI-2 sensor kinase/phosphatase LuxQ, phosphorelay protein LuxU, regulatory protein LuxO) probably involved in the export of AI-2 molecules. These genes are known to be probably its closing into AI-2 quorum sensing regulation^16^, while the regulatory mechanism remains unclear, and the level of proof is so far only genetic. Kozlova et al.^19^ demonstrated that the luxS isogenic mutant of *A. hydrophila* SSU enhanced biofilm formation and bacterial virulence in a septicemic mouse model and showed a decrease in swimming motility. In addition, the work of Cui et al.^97^, by constructing a *ΔluxS* mutant of *A. hydrophila* ATCC 7966, indicated that the AI-2 QS system upregulates biofilm formation and extracellular protease production. However, the regulatory mechanism of the AI-2 QS system in *A. hydrophila* remains unclear, and future studies are needed to investigate the detailed relationships between AI-2 system-associated genes and virulence factors.

#### Type 3 autoinducer (AI-3) system

Based on the genomic analysis, open reading frames for the *qseB* (GQR50_05730) and *qseC* (GQR50_05725) genes overlapping by 4 bp at the ATGA motif were found in the *A. hydrophila* HX-3 genome, which was consistent with the finding of Khajanchi et al.^20^. A promoter region with an SD sequence was also identified to be responsible for transcription of *qseBC* (Fig S3c). Compared with the Δ*qseB* mutant, in *A. hydrophila* SSU^20^, the AI-3 QS system enhances virulence, swarming and swimming motility and inhibits biofilm maturation. The study by Meng et al.^98^ suggested that biofilm formation and haemolytic activity were remarkably decreased in a △*qseC* mutant of *Aeromonas hydrophila* NJ-35 and showed no effect on motility, lipase activity or protease activity. In addition, interplay between AI-1 and AI-3 QS systems in *A. hydrophila* has already been demonstrated, and the transcription of the *qseBC* locus is negatively regulated by the AI-1 QS system^30^. While information on AI-3 metabolism in *A. hydrophila* is limited, future studies will address different functions of the AI-3 QS system in more detail.

## Conclusion

In summary, the complete genome of *A. hydrophila* HX-3 was sequenced and a total of 4318 CDSs were annotated. Comparative genomic analysis of HX-3 genome and 7 fully sequenced *A. hydrophila* strains revealed the core and pan genomes consisting of 3674 and 5778 genes, respectively. Analysis of putative virulence factors in comparison with the genomes of eight closely related *A. hydrophila* strains revealed conserved and unique virulence genes of this pathogenic species, including genes related to secretion systems, motility and adhesion, toxins, antibiotic and multidrug resistance, and iron acquisition, among others. In contrast, genes encoding lateral flagella and the synthesis and uptake of anguibactin were absent in all of the *A. hydrophila* strains. T3SS was not found in seven strains (with *A. hydrophila* AL06-06 being an exception). However, the Flp type IV pilus gene was missing only from the *A. hydrophila* HX-3 and AL06-06 genomes. In particular, the RTX toxin activator gene *rtxC* was absent in the five *A. hydrophila* genomes (D4, AL09-71, AL06-06, JBN2301, and NJ-35). In addition, genes related to the AI-1(*ahyI*, *ahyR*), AI-2 (*luxS*, *pfs*, *metEHK*, *litR*, *luxOQU*) and AI-3 (*qseB*, *qseC*) regulatory systems were identified to illustrate the comprehensive QS systems of *A. hydrophila* at the genetic level. Moreover, several new AHL compounds were detected by recombinant AhyI protein using UPLC-MS/MS analysis. Overall, the genomic information of *A. hydrophila* HX-3 provided with valuable data suggesting a relationship between pathogenicity and QS systems; and a framework for infection and the prevention of quorum sensing.

## Supplementary information

Supplementary Information 1.

Supplementary Information 2.

Supplementary Information 3.
