# Supplementary material for: Complete genome sequence of fish-pathogenic Aeromonas hydrophila HX-3 and a comparative analysis: insights into virulence factors and quorum sensing
Source: Sci Rep. 2020 Sep 23;10:15479. doi: 10.1038/s41598-020-72484-8 (PMC7512022; doi:10.1038/s41598-020-72484-8)
Supplement: Supplementary file 1 — Supplementary Information 1. [file 41598_2020_72484_MOESM1_ESM.docx]

**Complete genome sequence of fish-pathogenic *Aeromonas hydrophila* HX-3 and a comparative analysis: insights into virulence factors and quorum sensing**

Lei Jin ^a, b, c^, Yu Chen ^c, d^, Xiaojun Zhang ^c, d^, Zhaohui Qiao ^a, b^, Wenge Yang ^a, b, *^

^a^ College of Food and Pharmaceutical Sciences, Ningbo University, 315211, China
^b^ Key Laboratory of Animal Protein Food Deep Processing Technology of Zhejiang  Province, Ningbo University, Ningbo 315211, China

^c^ Marine Fishery Research Institute of Zhejiang Province, Zhoushan 316021, China

^d^ Zhoushan Fishery Environments & Aquatic Products Quality Monitoring Center of Ministry of Agriculture China, Zhoushan 316021, China

*Author for correspondence: E-mail, yangwenge_nbu@126.com

Tel, +86 0574-87604388

**Supplementary information**

**Supplementary Methods**

**Figure S1.** The mass spectra of ten putative AHL compounds produced by the recombinant AhyI protein expressed in *E. coli* BL21(DE3)/pET30a-*ahyI*.

**Figure S2.** Repeat elements in the genome of *A. hydrophila* HX-3.

**Figure S3.** (a) Genomic organization of AI-1 core genes *ahyI/R* and their flanking genes in strain HX-3 in comparison with other members of *A. hydrophila*. (b) A putative promoter and *lux* box in the 62 bp *ahyI/R* intergenic region. (c) Genetic organisation of the *qseBC* locus.

**Supplementary Methods:**

**Ultra performance liquid chromatography/tandem mass spectrometry (UPLC-MS/MS) analysis of AHLs.**

Identification of AHLs present in *E. coli* Rosetta (DE3)/pET30a-*ahyI* culture supernatant extracts was performed by an UPLC system (I-Class; ACQUITY) coupled to a triple quadrupole instrument (Xevo*^®^* TQ-S; Waters) equipped with an electrospray ionization (ESI) source. Chromatographic separation was obtained on a BEH-C18 column (1.7 μm, φ 2.1 ×100 mm; Waters) at 40 ℃. Mobile phases A and B were 0.1 % (v/v) acetic acid in water and 0.1% v/v acetic acid in methanol, respectively. A gradient elution was performed as follows: initially 60 % B, rising to 95 % B at 9 min, followed by a 12 min gradient to 60 % B using a flow rate of 0.2 mL/min. The column was equilibrated at 13 min using 60 % B. Samples were resuspended in 1 mL of methanol prior, and a 10 μL volume injected onto the column. The MS/MS spectrometric analyses were performed in the positive ion mode (ESI^+^) with a multiple reaction monitoring (MRM) system. The probe capillary voltage and a cone voltage were optimised at 2.5 kV and 36 V, respectively. Desolvation temperature was set at 500 ℃ with a cone gas flow of 150 L/h and a desolvation gas flow of 1000 L/h. Precursor ion scanning signals were recorded from m/z 50 to 500 Da, and product ions with a lactone ring were monitored at m/z 102 Da.

**Supplementary MS/MS spectra:**

**Figure S1.** The mass spectra of ten putative AHL compounds produced by the recombinant AhyI protein expressed in *E. coli* BL21(DE3)/pET30a-*ahyI*.

**Figure S2.** Repeat elements in the genome of *A. hydrophila* HX-3.

**Figure S3.** (a) Genomic organization of AI-1 core genes *ahyI/R* and their flanking genes in strain HX-3 in comparison with other members of *A. hydrophila*. Homologous gene domains are presented in the same colour and arrows indicate the relative orientations of the genes. The percentages indicate the identity of *ahyI/R* genes compared with homologous genes in *A. hydrophila* HX-3. (b) A putative promoter and *lux* box in the 62 bp *ahyI/R* intergenic region. The *lux* box and a putative promoter are highlighted in green and red, respectively. An SD sequence is highlighted in the underlined bases. (c) Genetic organization of the *qseBC* locus. The *qseBC* genes overlap with the ATGA motif. The promoter region (highlighted in red) is centred at -47.5 bp with respect to the start codon of *qseB*. An SD sequence is highlighted in the underlined bases.
